# Link N as a therapeutic agent for discogenic pain

**DOI:** 10.1002/jsp2.1008

**Published:** 2018-03-15

**Authors:** Hussain Noorwali, Michael P. Grant, Laura M. Epure, Padma Madiraju, Hee‐Jeong Sampen, John Antoniou, Fackson Mwale

**Affiliations:** ^1^ Division of Orthopaedic Surgery McGill University Montreal QC Canada; ^2^ SMBD‐Jewish General Hospital Lady Davis Institute for Medical Research Montreal QC Canada; ^3^ Division of Orthopaedic Surgery King Abdulaziz University Jeddah Saudi Arabia; ^4^ Department of Biochemistry Rush University Medical Center Chicago Illinois

**Keywords:** discogenic pain, intervertebral disc, Link N, low back pain

## Abstract

Neurotrophins (NTs) are the major contributors of sensory axonal sprouting, neural survival, regulation of nociceptive sensory neurons, inflammatory hyperalgesia, and neuropathic pain. Intervertebral disc (IVD) cells constitutively express NTs. Their expression is upregulated by proinflammatory cytokines present in the IVD during degeneration, which can promote peripheral nerve ingrowth and hyperinnervation, leading to discogenic pain. Currently, there are no targeted therapies that decrease hyperinnervation in degenerative disc disease. Link N is a naturally occurring peptide with a high regenerative potential in the IVD. Therefore, the suitability of Link N as a therapeutic peptide for suppressing NTs, which are known modulators and mediators of pain, was investigated. The aim of the present study is to determine the effect of Link N on NTs expression, nerve growth factor (NGF), brain‐derived neurotrophic factor (BDNF), and their cognate receptors TrkA and TrkB as they are directly correlated with symptomatic back pain. Furthermore, the neurotransmitter (substance P) was also evaluated in human annulus fibrosus (AF) cells stimulated with cytokines. Human AF cells isolated from normal IVDs were stimulated with interleukin‐1β (IL‐1β) and tumor necrosis factor‐α (TNF‐α) in the presence or absence of Link N. NGF release in the media was evaluated by Western blotting. Total RNA was isolated and gene expression was measured using real‐time PCR. Gene expression of NGF, BDNF, TrkA, and TrkB significantly decreased in human disc cells stimulated with either IL‐1β or TNF‐α supplemented with Link N when compared to the cells stimulated only with IL‐1β or TNF‐α. NGF protein expression was also suppressed in AF cells coincubated with Link N and IL‐1β when compared to the cells stimulated only with IL‐1β. Link N can suppress the stimulation of NGF, BDNF, and their receptors TrkA and TrkB in AF cells in an inflammatory milieu. Thus, coupled with previous observations, this suggests that administration of Link N has the potential to not only repair the discs in early stages of the disease but also suppress pain.

## 1 INTRODUCTION

The intervertebral discs (IVDs) are the main joints between two adjacent vertebrae within the spine. They are composed of the annulus fibrosus (AF), the central gelatinous nucleus pulposus (NP), and two endplates of hyaline cartilage that sandwich the AF and NP.^1^ In the adult human, the chondrocyte‐like NP cells are responsible for maintaining an extracellular matrix composed of randomly organized collagen fibrils and a high content of aggrecan.^2^ The fibroblast‐like AF cells are arranged parallel to the collagen fibers. Aging, poor nutrition, biomechanical,^3^, ^4^, ^5^, ^6^ biochemical,^7^, ^8^, ^9^, ^10^, ^11^ and genetic factors^12^, ^13^, ^14^, ^15^ are related to increased IVD degeneration. During degeneration, loss of proteoglycan content in the NP occurs, changing it from a gelatinous structure to a fibrotic texture as it becomes more collagenous, and fissures appear in both the NP and AF.^16^, ^17^

In normal human and animal IVDs, only the periphery of the AF is innervated by sensory and sympathetic perivascular nerve fibers.^18^, ^19^ It has been observed that the degenerative discs causing low back pain contain higher concentrations of sensory nerves in the endplates and NP compared with the painless degenerative IVDs.^20^, ^21^ This suggests that degeneration is commonly associated with back pain of IVD origin, possibly due to the increase in the number of nerve fibers in the IVD and loss of disc height.^22^ However, the mechanisms responsible for increased innervation of the IVD are unclear.

Currently, IVD hyperinnervation is often envisaged in terms of growth factors that are members of the NT family^23^, ^24^, ^25^ and “neurogenic” factors released by degenerative IVDs.^26^, ^27^ The density and distribution of sensory and sympathetic nerve fibers in peripheral tissues are dependent upon NTs for them to be able to survive through mechanisms that are not known, and the presence of growth factors will determine how many neurons will survive and the density of innervation.^28^ These molecules and their receptors have been observed locally in degenerative discs and from patients suffering from discogenic pain.^29^, ^30^ Among the family of NTs are nerve growth factor (NGF) and brain‐derived neurotrophic factor (BDNF), which exert their effects through a family of tyrosine kinase (Trk) receptors TrkA and TrkB, respectively.^31^ Disc cells are thought to be both a source and a target for NTs.^31^ Overexpression of NTs in the skin can lead to enhanced innervation and numbers of neurons in sensory ganglia.^32^ Conversely, in null mutants of NTs and their cognate receptors, most of the neurons are lost in the trigeminal ganglion, the primary sensory ganglion innervating the skin of the face and oral cavity.^33^ In the disc, hyperinnervation and NTs appear to play a key role in generating pain in the degenerative IVDs. The mechanisms by which neurons become reliant on NTs are not known. However, it has been shown that members of the bone morphogenetic protein (BMP) family can limit the population of neurons.

Although the AF is the main target of innervation of the discs, there are no targeted therapies that decrease hyperinnervation in degenerative disc disease. Link N is a 16‐amino acid, naturally occurring peptide, which represents the N‐terminal region of the link protein that stabilizes proteoglycan aggregates in both disc and cartilage, and is generated by MMPs during tissue turnover in vivo. We and others have previously shown the regenerative potential in the IVD.^34^, ^35^, ^36^, ^37^ Link N acts through the BMP type II receptor. Receptor activation leads to Smad1/5 signaling and an upregulation of BMP‐4 and BMP‐7 message levels.^38^

The purpose of this study was to investigate the effects of Link N on the expression of pain associated factors, NGF, BDNF, and substance P (SP), and their receptors in human AF cells. Our central hypothesis posits: (1) activation of NTs and neurotransmitters is the major driver of back pain transmission by inducing sensory neuronal plasticity (a peripheral mechanism) and spinal glial activity (a central mechanism); (2) the upregulation of BMP4 by Link N can decrease peripheral innervation; (3) and simultaneous inhibition of NTs and stimulation of matrix molecules by Link N will rescue patients from progression of disc pathology and associated pain.

## 2 MATERIALS AND METHODS

### 2.1 Human IVDs

Human lumbar spines were obtained through organ donation program in coordination with Héma‐Quebec from donors with a mean age of 42.8 years (range 32‐65 years). The spines were retrieved within 8 hours of cross‐clamping of carotids (official declaration of brain death) and the lumbar IVDs (*n* = 5 IVDs per spine) were separated from the adjoining vertebral body and morphologically graded according to Thompson grading system. Discs with calcification were not included in the study. All procedures were approved by the institutional review board of the Jewish General Hospital. After IVD isolation, the outermost layer of the AF was dissected and discarded, the AF region was separated from the NP region, and demarcation between the two regions was done based on the morphological structure. AF tissue was used for immunohistochemistry and protein expression.

### 2.2 Immunohistochemistry and staining

The AF tissue from human IVDs with degenerative grades 2 to 4 (*n* = 3 IVDs per grade) was fixed in Accustain (Sigma–Aldrich, St Louis, MO), paraffin embedded, and sectioned. Slides were incubated with anti‐NGF antibody (Cat. Number EP1320Y; Novus Biologicals, Littleton, CO) (0.1 μg/mL) overnight at 4°C. Secondary antibody and further processing of slides were performed using VECTASTAIN ABC kit (Vector Laboratories, Burlingame, CA) following manufacturer’s guidelines. Substrate and development were processed with 3,3′‐diaminobenzidine peroxidase substrate kit (Vector Laboratories, Burlingame, CA). Sections were counterstained with hematoxylin, dehydrated by sequential alcohol concentrations and xylene, and mounted in Permount (Thermo Fisher Scientific, Waltham, MA). Mouse IgG antibody (Cat. Number I‐2000; Vector Laboratories, Burlingame, CA) was used as negative control. Positive cells were validated based on immunohistochemistry reaction.

### 2.3 NGF protein expression in IVDs with different degenerative grades

The AF tissues from human IVDs with degenerative grades 2 to 4 (*n* = 3 IVDs per grade) were incubated in guanidine hydrochloride buffer (4 M guanidinium chloride, 50 mM sodium acetate, and 10 mM ethylenediaminetetraacetic acid) for 72 hours. NGF content was determined by Western blotting of extracts. Briefly, extracts were electrophoresed on 4%‐20% gradient gels (Bio‐Rad, Hercules, CA) and transferred to polyvinylidene fluoride (PVDF) membrane. Blots were probed with anti‐NGF antibody. Blots were developed by incubation with antirabbit immunoglobulin G antibody conjugated with horseradish peroxidase and Amersham ECL Prime chemiluminescent detection reagent (GE Healthcare, Piscataway, NJ). Images were captured on a VersaDoc molecular imager (Bio‐Rad, Hercules, CA).

### 2.4 Cultures of human AF cells

To evaluate the effect of Link N on interleukin‐1β (IL‐1β)‐induced NGF release in the media and IL‐1β‐ and tumor necrosis factor‐α (TNF‐α)**‐**induced NGF, BDNF, and SP gene expression, normal AF cells (PromoCell, Heidelberg, Germany) were expanded until Passage 3 using Dulbecco’s modified Eagle’s medium supplemented with 10% fetal bovine serum (Wisent Bioproducts, Montreal, Quebec, Canada) and antibiotics than seeded in 6‐well plates (2.5 × 10^5^ cells/well) and cultured until 80%‐90% confluence.

### 2.5 Effect of Link N on IL‐1β‐induced NGF release

At 90% confluency, AF cells were serum deprived overnight and incubated in culture medium containing either 1 μg/mL Link N, 10 ng/mL IL‐1β, or coexposed with Link N (1 μg/mL) and IL‐1β (10 ng/mL) for 72 hours. Unexposed cells were used as control. Conditioned media was collected 72 hours following treatment and electrophoresed on 4%‐20% gradient gels (Bio‐Rad, Hercules, CA). The separated proteins from the conditioned media were transferred on to PVDF membranes, and Western blotting was performed using antibodies recognizing NGF. The bound antibody was visualized by chemiluminescence. Images were captured on a Molecular Imager VersaDoc.

### 2.6 Effect of Link N on IL‐1β‐ and TNF‐α‐induced NGF, BDNF, and SP gene expression

AF cells were serum deprived overnight and exposed to either 10 ng/mL IL‐1β, 100 ng/mL TNF‐α, coexposed to IL‐1β 10 ng/mL with Link N 1 μg/mL or TNF 100 ng/mL with Link N 1 μg/mL for 48 hours. The expression level of NGF, BDNF, SP, and their receptors was quantified by real‐time polymerase chain reaction (RT‐PCR). After 48 hours, total RNA was extracted from AF cells using RNeasy kit (Qiagen, Hilden, Germany) following manufacturer’s instructions. One microgram of total RNA was reverse transcribed into cDNA (2 μL of cDNA solution) using the Omniscript Reverse Transcriptase kit (Qiagen, Hilden, Germany). Furthermore, the cDNA was mixed with 10 μL SYBR green, 1 μL primers (10 μm concentration), and RNAase free water to a final volume of 20 μL, and used per reaction well of RT‐PCR reactions using SYBR green chemistry run with 480 LightCycler Real Time System (Roche Applied Science, Penzberg, Upper Bavaria, Germany). Following reverse transcription, RT‐PCR was applied to quantitatively analyze message levels of NGF, BDNF, TAC1 (SP), and their receptors TrkA, TrkB, and TAC1R, respectively. The 18S ribosomal RNA was used as housekeeping gene. Gene expression was calculated using the ΔΔCt method. Initially, the expression of the target gene was normalized to 18S rRNA expression levels, and then the expression of the coincubations IL‐1β 10 ng/mL with Link N 1 μg/mL and TNF 100 ng/mL with Link N 1 μg/mL was normalized to IL‐1β 10 ng/mL and TNF 100 ng/mL incubation, respectively.

### 2.7 Statistical analysis

Data were analyzed by Analysis of Variance (ANOVA) followed by a post hoc Dunnett’s test. A *P*‐value of less than .05 was considered statistically significant.

## 3 RESULTS

**Figure 1:**
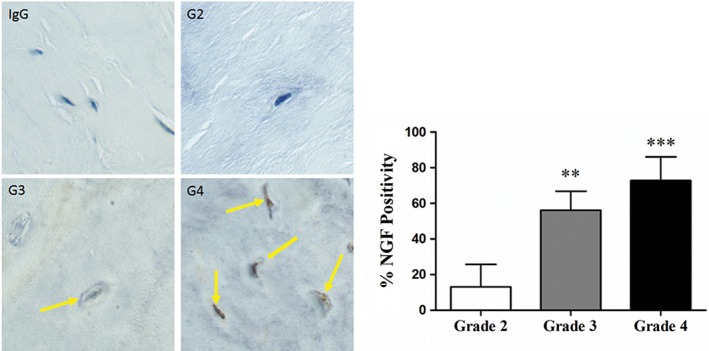
Expression of nerve growth factor (NGF) by immunohistochemistry in annulus fibrosus (AF) cells from Thompson grading intervertebral discs (IVDs). Representative images of AF cells from human discs grades 2 to 4 expressing NGF (arrows indicating the brown staining). IgG was used as a negative control. Bars represent means +/‐ SEMs; ANOVA post hoc Dunnett's, Grade 2 was used as the control. **, p < 0.01; ***, p < 0.001

**Figure 2:**
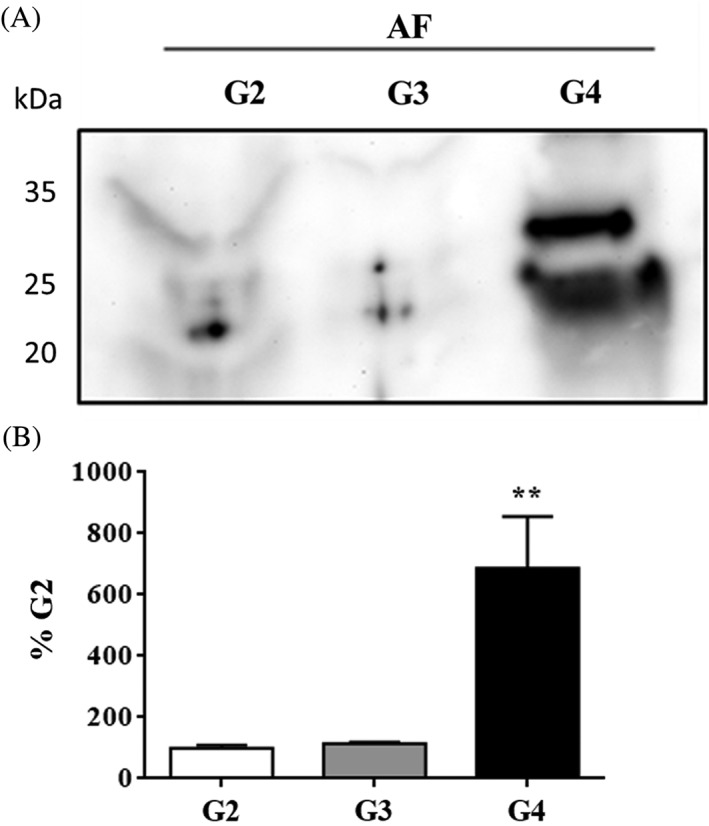
Analysis of newly synthesized nerve growth factor (NGF) in the discs. A, Western blot analysis of NGF in human discs from grades 2 to 4 from the annulus fibrosus (AF). B, Semiquantitative analysis of NGF. Means ± SEMs; *n* = 3. anova, post hoc Dunnetts’s; **P < .01

NGF is known to be a major contributor of sensory axonal sprouting, neural survival, regulation of nociceptive sensory neurons, inflammatory hyperalgesia, and neuropathic pain.^29^, ^30^, ^39^ To determine the expression of NGF with grade of degeneration in human AF cells, we performed immunohistochemistry on AF tissue and we assessed the NGF content by Western blotting on extracted NGF from AF tissue. The number of cells expressing NGF was higher in AF tissue from grade 4 (G4) discs compared with the AF cells from grade 3 (G3) and grade 2 (G2) discs (Figure 1). We also observe that in these donors, NGF in grade 4 (G4) AF tissue is enhanced by degeneration (Figure 2).

**Figure 3:**
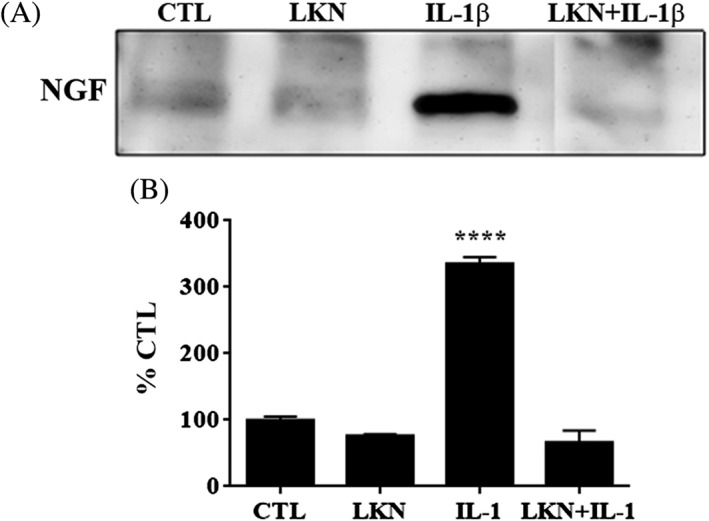
Analysis of nerve growth factor (NGF) released in the media. A, Western blot analysis of NGF protein released by annulus fibrosus (AF) degenerative cells exposed to Link N (LKN), interleukin‐1β (IL‐1β), or coexposed to Link N and IL‐1β (LKN + IL‐1β). B, Semiquantitative analysis of NGF. Means ± SEMs; *n* = 3. anova, post hoc Dunnetts’s; ****P < .0001

NGF can be upregulated by proinflammatory cytokines which are present in disc degeneration in vivo.^40^, ^41^, ^42^ To determine the effect of Link N on IL‐1β‐stimulated NGF release, normal AF cells were exposed to IL‐1β, Link N, or coexposed with Link N and IL‐1β. As expected, NGF release was upregulated when AF cells were incubated with IL‐1β (Figure 3, lane 3). However, NGF expression was suppressed in response to Link N in the presence of IL‐1β (Figure 3, lane 4) while Link N alone had no effect on NGF expression (Figure 3, lane 2).

**Figure 4:**
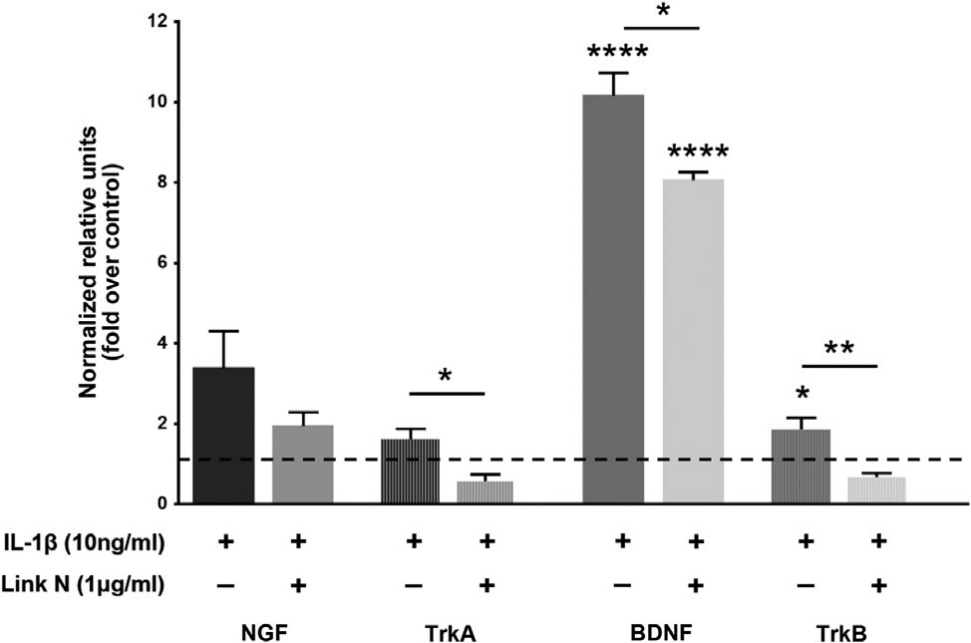
Effect of Link N on IL‐1β‐induced nerve growth factor (NGF), BDNF gene expression. Changes in NGF, TrkA, brain‐derived neurotrophic factor (BDNF), and TrkB gene expression in normal human annulus fibrosus (AF) cells after 48 hours exposure to Link N (1 μg/mL) + interleukin‐1β (IL‐1β) (10 ng/mL) or IL‐1β (10 ng/mL) alone. The results are shown as means ± SE (*n* = 3; anova, post hoc Dunnetts’s; *P < .05; **P < .01; ****P < .0001)

**Figure 5:**
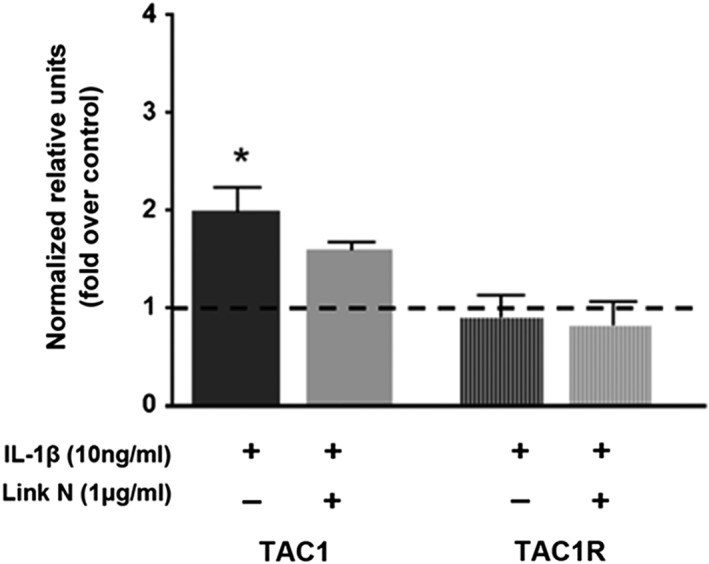
Effect of Link N on IL‐1β‐induced substance P (SP) gene expression. Changes in TAC1, and TAC1R gene expression in normal human annulus fibrosus (AF) cells after 48 hours exposure to Link N (1 μg/mL) + interleukin‐1β (IL‐1β) (10 ng/mL) or IL‐1β (10 ng/mL) alone supplementation. The results are shown as means ± SE (*n* = 3; anova, post hoc Dunnetts’s; *P < .05; ***P < .001)

In view of previous results, where NGF, a member of the NT family known to be involved in IVD hyperinnervation, was suppressed by Link N, and with the understanding that IL‐1β can also stimulate other neurotrophic factors such as BDNF and/or neurotransmitters SP,^30^, ^43^ we decided to determine the effect of Link N on NGF, BDNF, SP, and their receptors. Thus, human AF cells were coexposed to Link N and IL‐1β for 48 hours and relative gene expression was evaluated for NGF, BDNF, SP (TAC1), and their receptors TrkA, TrkB, and TAC1R, respectively. Results are expressed relative to cells unexposed to Link N (Figures 4 and 5). As expected, IL‐1β increased significantly the expression of BDNF (*P* < .0001) and TrkB (*P* = .0325) when compared to their controls (Figure 4). The coexposure with IL‐1β and Link N led to a significant decrease in BDNF (*P* < .0127) and TrkB (*P* < .0077) gene expression in AF cells when compared with controls (Figure 3). However, no significant effect was observed on TAC1 and TAC1R expression when Link N was coincubated with IL‐1β (Figure 5).

**Figure 6:**
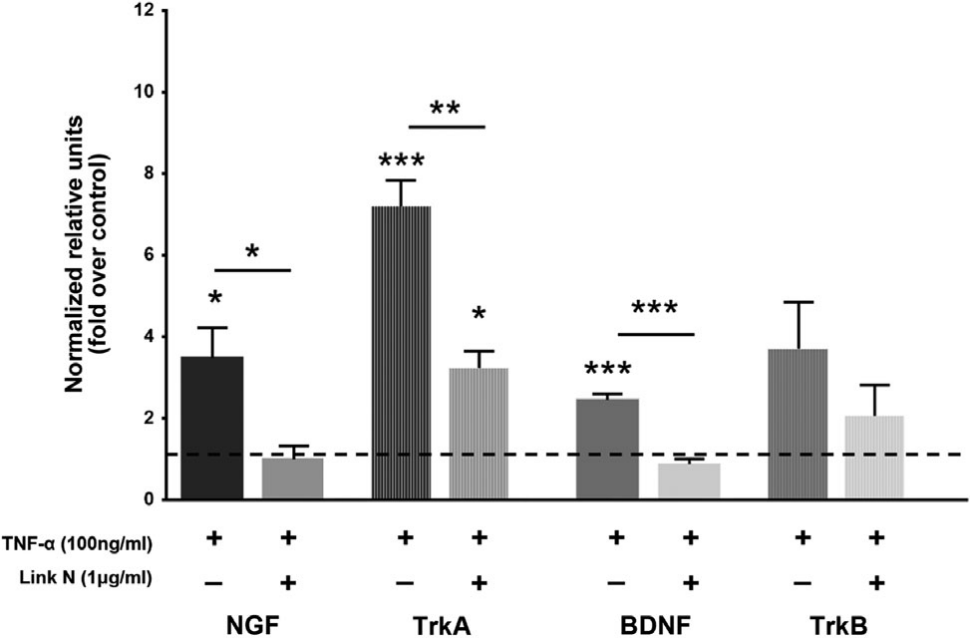
Effect of Link N on tumor necrosis factor‐α (TNF‐α)‐induced nerve growth factor (NGF), BDNF gene expression. Changes in NGF, TrkA, brain‐derived neurotrophic factor (BDNF), and TrkB gene expression in normal human annulus fibrosus (AF) cells after 48 hours exposure to Link N (1 μg/mL) + TNF‐α (100 ng/mL) or TNF‐α (100 ng/mL) alone. The results are shown as means ± SE (*n* = 3; anova, post hoc Dunnetts’s; *P < .05; **P < .01; ***P < .001)

**Figure 7:**
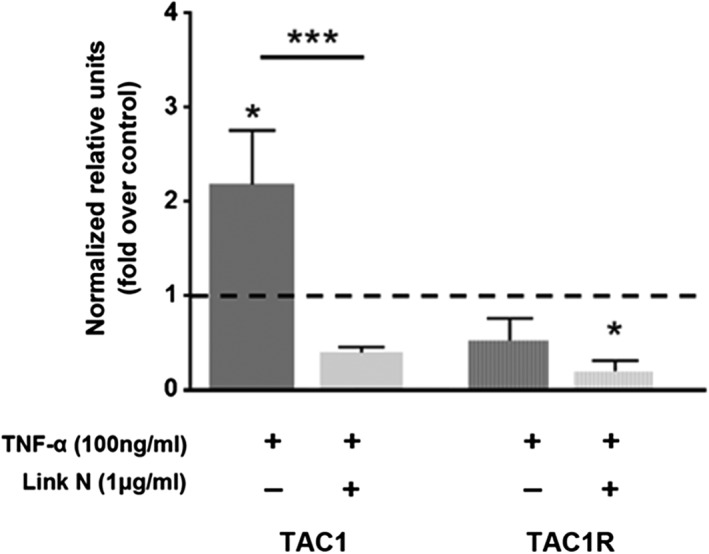
Effect of Link N on tumor necrosis factor‐α (TNF‐α)‐induced substance P (SP) gene expression. Changes in TAC1 and TAC1R gene expression in normal human annulus fibrosus (AF) cells after 48 hours exposure to Link N (1 μg/mL) + TNF‐α (100 ng/mL) or TNF‐α (100 ng/mL) alone supplementation. The results are shown as means ± SE (*n* = 3; anova, post hoc Dunnetts’s; *P < .05; ***P < .001)

The neurotrophic factors and/or neurotransmitters can also be upregulated by TNF‐α.^41^ To investigate this, human AF cells were exposed to Link N for 48 hours in the presence of TNF‐α, and relative gene expression was evaluated for NGF, BDNF, SP, and their receptors (Figures 6 and 7). As expected, TNF‐α increased significantly the gene expression of neurotrophic factors (*P* = .0146 for NGF, *P* = .0002 for BDNF), SP (TAC1, *P* = .0130), and NGF receptor (*P* = .0001) when compared to their controls. Our results demonstrate that Link N could significantly downregulate the gene expression of NGF (*P* = .0216), BDNF (*P* = .0001), TAC1 (*P* = .0008), and of NGF receptor TrkA (*P* = .0016) in AF cells. A decrease in TrkB expression was also observed, although it did not reach statistical significance.

## 4 DISCUSSION

During disc degeneration, it has been demonstrated that IVD cells secrete proinflammatory cytokines such as TNF‐α, IL‐1α, IL‐1β, IL‐6, and IL‐17. These cytokines in addition to promoting the degradation of the IVD extracellular matrix, trigger the secretion of NGF and SP.^30^, ^31^, ^40^, ^43^

NGF is known to be a major contributor to sprouting of sensory axons, neural survival, regulation of nociceptive sensory neurons, inflammatory hyperalgesia, and neuropathic pain. The question whether NGF is expressed in different parts of normal IVD and its relationship to disc degeneration has been widely discussed.^29^, ^30^, ^39^ It is thought that enhanced expression of NGF is then transported to the dorsal root ganglion where it acts on TrkA‐expressing neurons. This leads to the stimulation of peptides that mediate pain, such as SP and calcitonin gene‐related peptide. The consequence of increased levels of NGF in the IVD, as well as the depletion of proteoglycan, is ingrowth of nociceptive nerve fibers and pain.

We and others have previously demonstrated that Link N can act as an anabolic agent in the disc enhancing proteoglycan synthesis and depleting proteinase expression.^34^, ^35^, ^36^, ^37^, ^38^, ^44^, ^45^, ^48^, ^52^, ^53^, ^54^, ^55^, ^56^, ^57^, ^58^, ^59^, ^60^ We have shown that Link N can maintain its anabolic effects in an inflammatory milieu when human IVD cells were exposed to IL‐1.^36^ Using a rabbit model of IVD degeneration, we have generated data indicative of the role of Link N and Short Link N, a recently discovered fragment of the Link N peptide, in disc repair.^31^, ^45^ Interestingly, in a recent report by Yeh et al, the regenerative potential of Link N can be further enhanced when in combination with the antioxidant fullerol.^44^ However, the role of Link N in regulating the effects of the inflammatory cytokines in discogenic pain remains unknown.

Among the molecules that are involved in nerve growth and hyperinnervation of pathological IVDs are some members of the family of NTs. As previously demonstrated by Purmessur et al, we found significant increases in the number of AF cells expressing NGF with grade of degeneration.^30^ In addition to the number of cells, total content of NGF in AF tissue was increased in severely degenerative discs (grade 4). Although the synthesis of NGF can be induced by the exposure of AF cells to IL‐1,^46^ we demonstrate that Link N can inhibit this response. In addition to the expression of NGF, Link N was also capable of inhibiting the upregulation of TrkA, BDNF, and TrkB induced by IL‐1 beta. Link N decreased the expression of SP (TAC1), and the receptor for SP, TAC1R, nociceptive factors shown to be upregulated by inflammatory cytokines in IVD cells.^47^ The inflammatory cytokine, TNF‐α, has also been purported to upregulate factors associated with discogenic pain in IVD cells.^46^ Our data indicate that Link N can inhibit the upregulation of NTs and their receptors in human AF cells following TNF‐α exposure.

Recently, it has been demonstrated that Link N can stimulate the BMP type II receptor activating Smad1/5 signaling and upregulating BMP4 in IVD cells.^38^, ^48^ BMP4 has been demonstrated to decrease peripheral innervation in an animal model.^49^ Therefore, one mechanism for the ability of Link N to inhibit the expression of NTs associated with pain in the IVD is by activating the BMP signaling pathway.

Another mechanism by which Link N may be inhibiting the induction of NTs by inflammatory cytokines in IVD cells is by decreasing cytokine signaling pathway. In our unpublished work, human chondrocytes coexposed to IL‐1β and Link N demonstrated a reduced activation of nuclear factor‐κB when compared to chondrocytes treated with IL‐1β alone.^50^ Since the IL‐1β signaling pathway is important in mediating NTs upregulation, factors that inhibit IL‐1β receptor signaling (ie, IL‐1 receptor antagonist) may play a role in regulating the pathogenesis of degenerative IVD disease.

The results of the present study demonstrate a potential important mechanism by which pain in human IVDs may be initiated. When stimulated by IL‐1β or TNF‐α isolated human disc cells upregulated the expression of NTs and their receptors while injured bovine discs stimulated SP. Furthermore, our results demonstrate that Link N can suppress IL‐1β and TNF‐α stimulated NTs and their receptors expression as well as SP. To our knowledge, the present study is the first to demonstrate a mechanism by which Link N may play an important role in the suppression of pain markers involved in IVD degeneration. Additionally, our results may have important future implications in the treatment of discogenic pain.

One limitation of the present study is that human AF cells were cultured in monolayer. It has been demonstrated that monolayer cultured AF cells can assume a flattened, spindle‐shaped morphology and loose the phenotype. Three‐dimensional culture results in a rounded cell phenotype and increased proteoglycan synthesis compared to cells grown in monolayer. In spite of this limitation, the present study has provided insight into the potential for targeted pain intervention by Link N, which may facilitate clinical trial design. Interestingly, there was no significant effect on either NGF (Figure 3, lane 2) or TAC1 and TAC1R expression when Link N was incubated alone (Figure 5). This suggests that Link N would have no effect on nociceptive nerve ingrowth into the disc IVD and associated pain.

Recently, we modeled discogenic pain in a slowly progressive surgical mouse model of discogenic low back pain, induced by stabbing the lumbar discs (L4/5, L5/6, and L6/S1) and removing a part of the NP^51^ and monitor pain‐related behaviors longitudinally over 12 weeks. Future studies will determine the effect of Link N on pain in this mouse model by either administering Link N prophylactically starting at time of surgery for 8 weeks or whether the disease can be modulated when targeted therapeutically. These studies will help to address some key questions as to (1) whether affecting disc pathology will modulate plasticity changes in the pain pathway and (2) whether there are optimal times during the disease process to achieve this effect.

In Table 1, we show that Link N is an ideal IVD target profile based on the present study and other published work.^35^, ^36^, ^37^, ^45^, ^52^, ^53^, ^54^, ^55^, ^56^, ^57^, ^58^, ^59^, ^60^ In Table 2, a summary of the clinical and market opportunities for Link N is presented.

**Table 1:** Link N is an ideal intervertebral disc (IVD) target profile

Product element	Link N	Anelgesic/anti‐inflammatory	Steroids	Biologics
Increase disc/matrix synthesis	**√**	X	X	**√**
Decrease disc degeneration	**√**	X	X	**√**
Reduce inflammation	**√**	**√**	**√**	**√**
Active in inflammatory environment	**√**	**√**	**√**	**√**
Reduce pain	**√**	**√**	**√**	**√**
Nonosteogenic	**√**	**√**	**√**	X
Lost lasting effects	**√**	X	X	**√**
Limited or no long‐term side effects	**√**	X	X	?
Cost effective	**√**	**√**	**√**	X

Ideal characteristics of an IVD therapeutic.

**Table 2:** Clinical scenario in which Link N can be used

*Adjunct to disc herniation surgery*
Discectomies
Removal of bulging disc material relieves pain but does not encourage regeneration/repair
Younger population treatment, earlier stage, prone to further degeneration if no repair is induced
Spinal fusions
Performed at advanced levels of disc degeneration
Often leads to further degeneration in adjacent discs by altering biomechanics/kinematics
*Prophylaxis to avoid surgery*
In conjunction with/replacement for steroid injections
Known short‐term benefits of steroids, but long‐term effectiveness was less established
Steroids may address acute inflammation and allow the body’s normal healing process to lead to long‐term improvement, but do not cause disc repair

The fact that NGF positivity was highest in Thompson grade IV discs raises the question of whether Link N would work to relieve pain in patients with early stage degenerative disc disease, considering that NGF expression might be significantly lower compared to Thompson grade IV or V. We have no reason to believe that it would not work as in earlier work with painful IVD excised from patients with back pain, it was found that only painful IVD expressed NGF produced by microvessels which come to populate the normally avascular IVD.^29^ In the present study, although the levels of NGF expression were lower than those produced by grade IV discs, NGF blockade would be highly efficacious as recent clinical trials for knee osteoarthritis pain indicated.^61^ Intradiscal injection (using a small gauge needle) would be a favored method for delivering Link N to get desired effects quickly and directly. Based on our previous studies and since the disc has no blood vessels, Link N would likely be diffused very slowly at a sustained rate.

In this study, we chose to examine the effects of Link N on proinflammatory‐mediated regulation of NTs in the AF only because these cells were readily available and we were not sure if Link would have an effect or not. However, NP cells are also known to express the NTs NGF and BDNF. Thus, future studies will look at NP cells to see whether the same effects could be observed. The mechanisms by which Link N inhibits proinflammatory‐mediated upregulation of NTs and SP are unclear. Link N acts through the BMP receptor and augments the effects of endogenous BMPs.^38^ It is therefore possible that inhibition of proinflammatory‐mediated upregulation of NTs and SP occurs indirectly because of BMP‐mediated changes in the disc.

In summary, our study demonstrates that Link N has the ability to suppress the NTs, NGF, and BDNF, as well as their receptors TrkA and TrkB. Link N was also able to suppress SP and its receptor TAC1R. These are features needed for any agent designed to suppress pain factors. Therefore, in principle, Link N supplementation could be a viable option for treating discogenic pain. Our results support the concept that the administration of Link N has therapeutic potential for pain relief.
